# Probable Three-Species In Vivo Transfer of *bla*_NDM-1_ in a Single Patient in Greece: Occurrence of NDM-1-Producing *Klebsiella pneumoniae*, *Proteus mirabilis*, and *Morganella morganii*

**DOI:** 10.3390/antibiotics12071206

**Published:** 2023-07-20

**Authors:** Georgios Meletis, Andigoni Malousi, Areti Tychala, Angeliki Kassomenaki, Nikoletta Vlachodimou, Paraskevi Mantzana, Simeon Metallidis, Lemonia Skoura, Efthymia Protonotariou

**Affiliations:** 1 Department of Microbiology, AHEPA University Hospital, School of Medicine, Aristotle University of Thessaloniki, 54636 Thessaloniki, Greece; aretich@gmail.com (A.T.); angelikikasso@gmail.com (A.K.); nikoletta.vlachodimou@gmail.com (N.V.); vimantzana@gmail.com (P.M.); mollyskoura@gmail.com (L.S.); protonotariou@gmail.com (E.P.); 2 Laboratory of Biological Chemistry, School of Medicine, Aristotle University of Thessaloniki, 54124 Thessaloniki, Greece; andigoni@auth.gr; 3 First Department of Internal Medicine, Infectious Diseases Division, AHEPA University Hospital, School of Medicine, Aristotle University of Thessaloniki, 54636 Thessaloniki, Greece; metallidissimeon@yahoo.gr

**Keywords:** *Klebsiella pneumoniae*, carbapenemases, NDM, plasmid, *Proteus mirabilis*, *Morganella morganii*

## Abstract

NDM carbapenemase-encoding genes disseminate commonly among Enterobacterales through transferable plasmids carrying additional resistance determinants. Apart from the intra-species dissemination, the inter-species exchange of plasmids seems to play an additional important role in the spread of *bla*_NDM_. We here present the genetics related to the isolation of three species (*Klebsiella pneumoniae*, *Proteus mirabilis*, and *Morganella morganii*) harboring the *bla*_NDM-1_ gene from a single patient in Greece. Bacterial identification and antimicrobial susceptibility testing were performed using the Vitek2. Whole genome sequencing and bioinformatic tools were used to identify resistance genes and plasmids. *Bla*_NDM-1_ harboring plasmids were found in all three isolates. Moreover, the plasmid constructs of the respective incomplete or circular contigs showed that the *bla*_NDM-1_ and its neighboring genes form a cluster that was found in all isolates. Our microbiological findings, together with the patient’s history, suggest the in vivo transfer of the *bla*_NDM-1_-containing cluster through three different species in a single patient.

## 1. Introduction

The emergence and spread of carbapenem-resistant Gram-negative nosocomial pathogens, especially those producing one or more of the major carbapenemases, are of great concern to public health [1]. Carbapenemases are enzymes that are able to hydrolyze carbapenems and the majority of β-lactams. *Klebsiella pneumoniae* carbapenemase (KPC), Verona integron-encoded metallo-β-lactamase (VIM), Imipenemase (IMP), Oxacillinase-48 (OXA-48), and the later New Delhi metallo-β-lactamase (NDM) are clinically the most important carbapenemases because they often confer high-level resistance to carbapenems and have successfully spread since they are mainly located on a variety of plasmids [2].

The existence of plasmids harboring resistance genes, initially called R-factors, has been known for decades, and nowadays, the correlation of resistance plasmids with the spread of resistance genes to new organisms is widely accepted. More light was shed on the understanding of antimicrobial resistance dissemination with the later discovery of smaller and more versatile mobile genetic elements such as transposons and gene cassettes accompanied by integrons. Using such elements as vehicles, resistance genes are able to relocate among different DNA molecules, including the bacterial chromosome and various plasmids. In such cases, and when the plasmids are conjugative or mobilizable, resistance genes can be transferred to other bacterial cells of the same, and in some cases, of different species.

In particular for NDMs, their extensive dissemination among different Enterobacterales is mainly due to the presence of the *bla*_NDM_ gene in transferable plasmids of various incompatibility groups that propagate within each species, including incompatibility group A/C-type (IncA/C-type), incompatibility group N (IncN), and incompatibility group F (IncF) types [3,4,5]. Moreover, these plasmids commonly carry additional resistance determinants to antimicrobial categories other than the β-lactams, thus contributing to multi-drug resistance (MDR), leaving very few therapeutic options. Interspecies dissemination is deemed to play an important role as well, even though it is not frequently documented. At the clinical level, the presence of NDM is important for yet another reason. New antibiotic combinations with novel β-lactamase inhibitors such as ceftazidime/avibactam and meropenem/vaborbactam have been made available lately. Unfortunately, however, these are not active for a specific category of carbapenemases called the metallo-β-lactamases. NDM enzymes, together with VIM and IMP, belong to this category for which no clinically useful inhibitors are available for the time being. Due to the aforementioned reasons, and since carbapenems are the safest last line treatment option for Gram-negative bacterial infections, the detection of NDM in hospital settings is considered of high importance, and measures are needed to tackle its dissemination.

This study reports the isolation of three *bla*_NDM-1_–harboring Enterobacterales (*Klebsiella pneumoniae*, *Proteus mirabilis*, and *Morganella morganii*) from a single patient in Greece together with whole genome sequencing analysis that suggest in vivo transfer of the NDM-1-encoding gene.

## 2. Results

### 2.1. Patient History

In April 2021, a 67-year-old male was admitted to AHEPA University Hospital (Thessaloniki, Greece) due to fever, cough, and shortness of breath. After testing positive for severe acute respiratory syndrome coronavirus 2 (SARS-CoV-2) by PCR, the patient was hospitalized for coronavirus infectious disease 2019 (COVID-19). From his medical record, the patient had a history of pre-existing and well-managed type-2 diabetes mellitus. During his hospitalization, fecal surveillance samples taken for infection control purposes identified a carbapenem-resistant (CR) *K. pneumoniae*. The patient’s clinical condition soon deteriorated, and he was transferred to the intensive care unit (ICU) where the patient was intubated. During the ICU stay, the patient was additionally colonized with an extensively drug-resistant *Acinetobacter baumannii* strain. The patient underwent a series of co-infections during the hospitalization in the ICU. These included a carbapenem-resistant *K. pneumoniae* blood infection (isolate D730) on 5 May 2021 and *P. mirabilis* and *A. baumannii* lower respiratory tract infections on May 5 and 10, respectively. Susceptibility testing was performed upon isolation for each strain. The patient’s treatment included colistin, tigecycline, ceftazidime/avibactam, and aminoglycosides. The patient was discharged from the ICU on 14 July 2021, hospitalized in the Internal Medicine Department, and left the hospital on 19 July 2021.

A few months later, however, on 8 October 2021, he was again admitted to the hospital presenting with respiratory infection and final stage renal disease. Blood cultures taken upon admission gave positive results for a carbapenem-resistant *P. mirabilis* (isolate D1633) and a carbapenem-resistant *M. morganii* (isolate D1644). CR *P. mirabilis* was also recovered from the patient’s urine culture. In the following days the patient’s clinical condition deteriorated, necessitating his transfer to the ICU. On the seventh day of his hospitalization, blood cultures were repeated and showed a simultaneous infection by CR *P. mirabilis*, CR *M. morganii*, and *Enterococcus faecium*. The patient went into septic shock and eventually died of cardiac arrest on 15 October 2021.

### 2.2. Susceptibility Testing

All three study isolates were multi-drug-resistant, presenting high minimum inhibitory concentrations (MICs) to β-lactams, including carbapenems (Table 1).

### 2.3. Genomic Characterization of the Isolates

Whole genome sequencing (WGS) was performed on the three isolates using the same sequencing Ion Torrent protocols and data analysis pipelines. The main WGS summary statistics are shown in Table 2. Overall, 0.7–1.7 M reads per isolate were produced. The read length was normally distributed across isolates with an average 305–311 bp and the duplication rate was 13.2%. The assembled genomes contained on average 90 contigs for *P. mirabilis* and *M. morganii. K. pneumoniae* assembly was significantly more fragmented due to the 35% larger genome size, yet the N50 value was similar to that of *P. mirabilis*.

### 2.4. Taxonomic Assignment

The taxonomic classification of the isolates showed that the abundance of the *P. mirabilis, M. morganii*, and *K. pneumoniae* species was on average 77% among other bacteria (Table 2). Taxonomic assignments to *E. coli*-like sequences covered 7% of the total bacteria-derived *P. mirabilis* and *M. morganii* sequencing reads and 3% of the *K. pneumoniae*. Half of the *M. morganii* sequencing reads belonged to *morganii* subspecies, while the remaining did not have any subspecies assignments. *P. mirabilis* sequencing reads were further taxonomically assigned to the HI4320 (10% of the reads) and BB2000 (17% of the reads) strains, while the remaining had no further strain assignment. *K. pneumoniae* isolate was taxonomically assigned to *pneumoniae* subspecies (40% of the reads), while 54% of the *K. pneumoniae* reads had further taxonomic assignment. The remaining 6% classified to a wide range of *K. pneumoniae* strains and subspecies, including rhinoscleromatis SB3432 (*subsp*), *K. pneumoniae* ozaenae (*subsp*), *K. pneumoniae* MGH 39 (*str*), and *K. pneumoniae* 500_1420 (*str*).

### 2.5. Antimicrobial Resistance Genes

**Figure 1:**
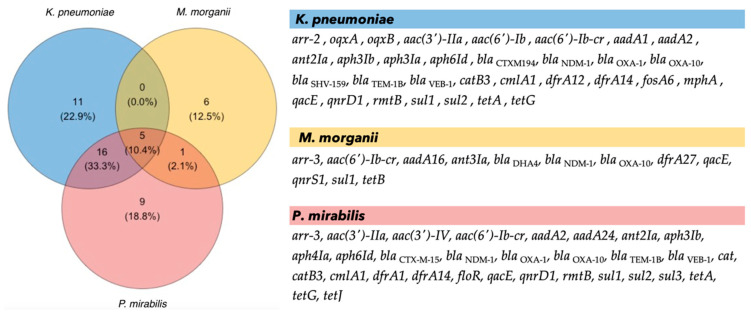
Number of shared (**left**) and list of (**right**) AMR genes detected in each isolate.

In silico analysis detected 48 antimicrobial resistance (AMR) genes (>98% identity). *K. pneumoniae* and *P. mirabilis* carried 16 common AMR genes that corresponded to almost half of the total genes (Figure 1). *OqxAB* genes conferring resistance to fluoroquinolones were detected in *K. pneumoniae*. *M. morganii* included 12 AMR genes. Among these, *aac(6’)-Ib-cr*, *bla*_NDM-1_, *bla*_OXA-10_, *qacE*, and *sul1* were shared among all isolates.

### 2.6. Replicon Types of Plasmids

The replicon types of the detected plasmids, according to PlasmidFinder, are shown in Table 3. The Inc plasmid family is very frequent in Enterobacterales. In this study, Inc plasmids were detected in all isolates in a single or in multiple subtypes. lncC and IncFIA(HI1) types were found in *K. pneumoniae* and *P. mirabilis* isolates. *M. morganii* included an IncN plasmid subtype. *bla*_NDM-1_ was identified in IncFIA(HI1) replicons for the *K. pneumoniae* and *P. mirabilis* isolates. The initial gene detection analysis of the IncN replicon type in *M. morganii* did not verify the presence of *bla*_NDM-1_ in the assembled 6.5 kb contig. Further analysis of the assembled *M. morganii* genome using MOB-suite revealed the presence of *bla*_NDM-1_ in a plasmid sequence that is classified to the AA552 plasmid type and that is identical to the closest cluster type of the IncN replicon. *M. morganii* includes a relaxase of the MOB_F_ type in a plasmid contig that is of the same AA552 cluster type. Both *K. pneumoniae* and *P. mirabilis* carried a relaxase of the MOB_H_ family on the IncC replicon. Overall, relaxases are essential components for conjugative DNA transfer, and both MOB_F_ and MOB_H_ types are prevalent in transmissible plasmids hosted in γ-Proteobacteria.

### 2.7. Construction and Comparative Analysis of the Plasmids Harboring bla_NDM-1_

**Figure 2:**
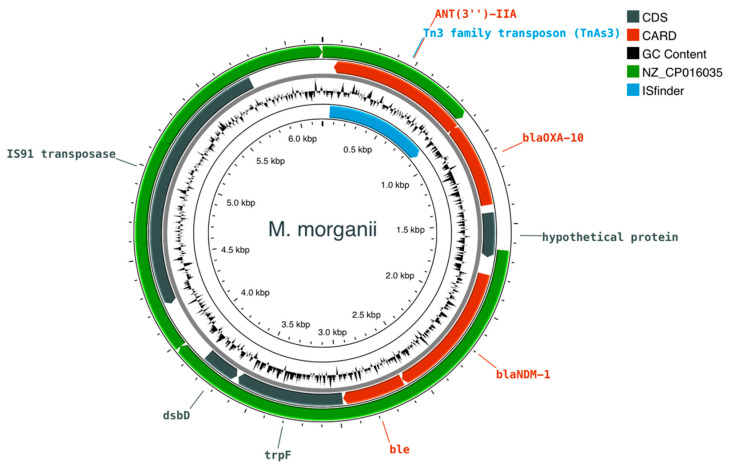
Circular map of the *bla*_NDM-1_-containing plasmid of *M. morganii*. Features colored in red correspond to AMR genes identified by CARD. Grey colored tracks correspond to additional coding regions other than AMR genes, of the plasmid (CDS). The green track shows the homology level with the MOB_F_ (NZ_CP016035) oriT type. The hypothetical protein on the left side of the plasmid belongs to an integration/excision mobile element.

Plasmids that carry *bla*_NDM-1_ were further analyzed in order to build the topological features of the plasmid constructs and to assess the level of gene co-localization. Overall, the plasmid constructs of the respective incomplete contigs showed that *bla*_NDM-1_ and its neighboring genes are found in all isolates. Table 4 lists relevant genomic features of the plasmids harboring the *bla*_NDM-1_ gene, including the confidence level of being a plasmid and respective cluster group, the putative origin of transfer (oriT) sequences, and distance of the nearest neighbor. During the conjugation process, oriT sequences are recognized by relaxases and initiate the transfer. *oriT* sequences of MOB_F_ type (NZ_CP016035) were found in both *K. pneumoniae* and *P. mirabilis*. In *M. morganii* no oriT sequences are identified; however, a strong homology with the same MOB_F_ type is detected, as shown in the corresponding track in Figure 2. Notably, all isolates lacked any membrane-associated mating pair formation (MPF) complex, implying the presence of mobilizable *bla*_NDM-1_-containing plasmids for *K. pneumoniae* and *P. mirabilis*. Overall, all *bla*_NDM-1_ plasmids were identified as incomplete.

**Figure 3:**
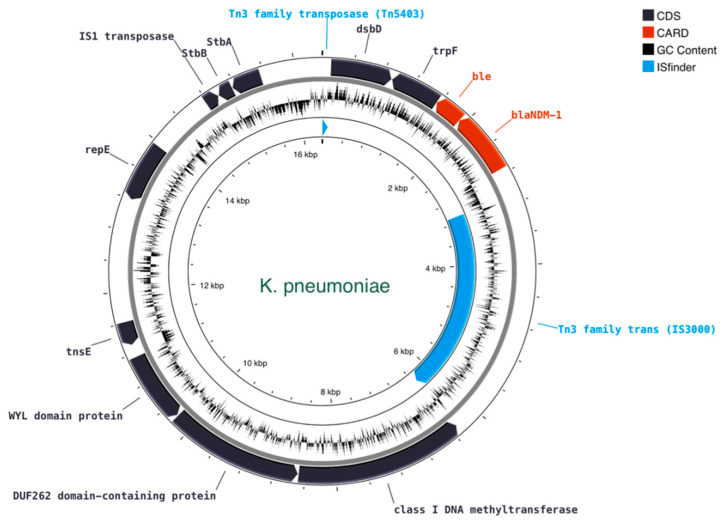
Circular map of the *bla*_NDM-1_-containing plasmid of *K. pneumoniae*. Features colored in red correspond to AMR genes identified by CARD.

*K. pneumoniae* (ST11) was the most abundant isolate in plasmids and AMR genes (Figure 1, Table 3). The *bla*_NDM-1_ was found on a mobilizable 16,354 bp plasmid with a MOB_F_ oriT type closest to *K. pneumoniae* (Figure 3). *bla*_NDM-1_ colocalized with *ble*, and both were enclosed by the *trpF* and *dsbD* genes on the 3′ end, and by the *Tn3* family transposase element on the 5′ end.

**Figure 4:**
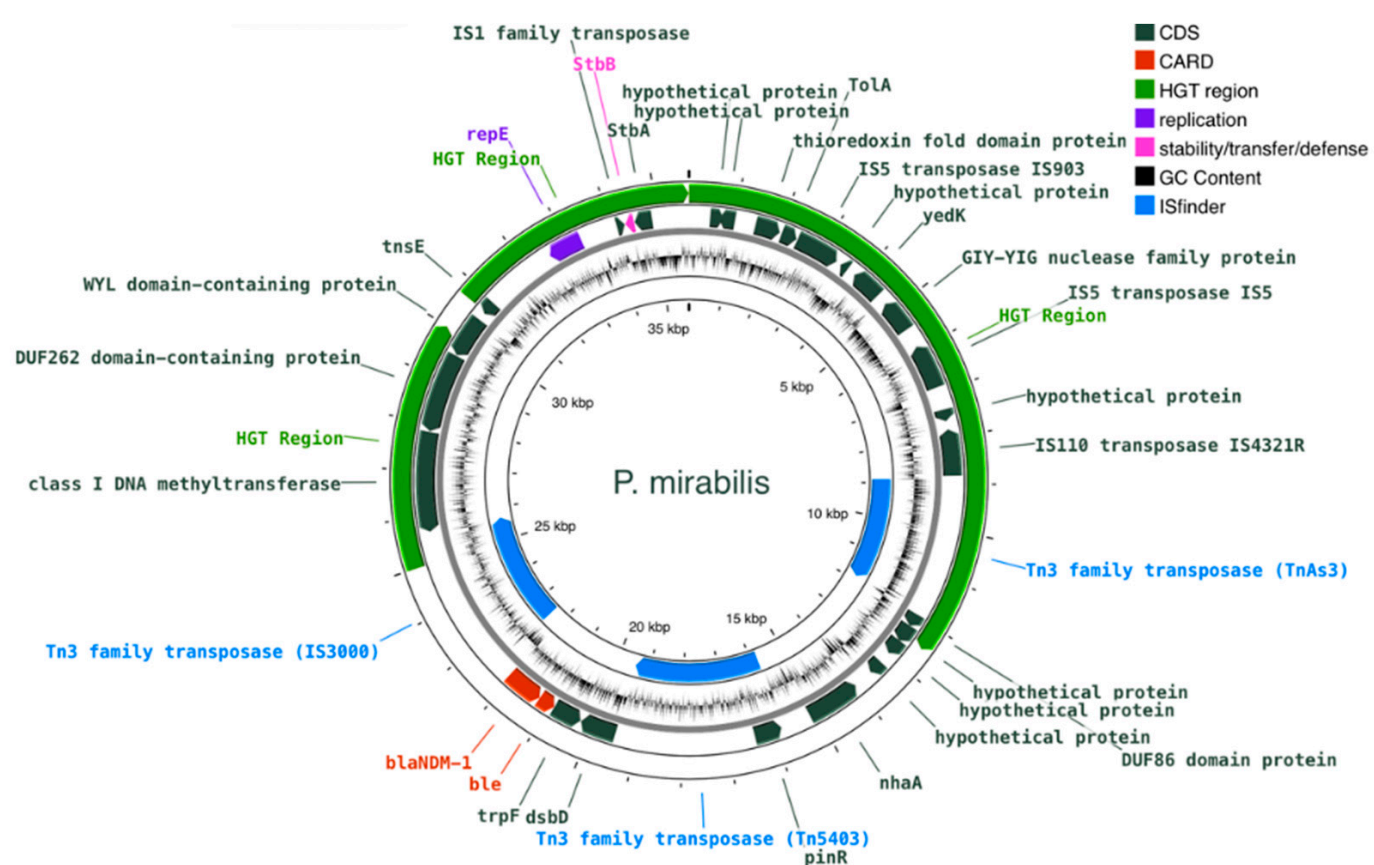
Circular map of the *bla*_NDM-1_-containing plasmid of *P. mirabilis*. Features colored in red correspond to AMR genes identified by CARD. The HGT region colored in green corresponds to a horizontal gene transfer region.

Similar organizational features are present on the *bla*_NDM-1_-containing plasmid *P. mirabilis* isolate. The genome of *P. mirabilis* includes a mobilizable plasmid of 35,703 bp (57.54× depth of coverage) that contains *bla*_NDM-1_ and is identified as being closest to *K. pneumoniae* (MOB_F_, MASH: 0.006). The plasmid was identified by both Deeplasmid and plasmidSPAdes (Figure 4).

In *M. morganii*, the *bla*_NDM-1_ was identified in a 6191 bp contig (162× depth) that was characterized as a plasmid-deriving sequence by both PlasmidSPAdes and MOB-suite. AA552 is the closest cluster type of the *bla*_NDM-1_-containing plasmid, which is also the closest cluster type of the IncN replicon detected in the *M. morganii* isolate (Table 3). Besides *bla*_NDM-1_, the plasmid contains three other AMR genes, *bla*_OXA-10_, *ant(3″)-IIa*, and *ble* (Figure 2). In addition, the plasmid includes a mobile genetic element that is involved in integration/excision mechanisms (WP_000050481.1, mobileOG_000731050). Moreover, in all three isolates, the best scoring matches (e-value = 0) of the *bla*_NDM-1_-containing plasmids with the insertion sequences of the ISfinder database belong to the Tn3 family of replicative transposons. The sequences producing high-confidence alignments are shared by at least two isolates, and include IS3000 (*K. pneumoniae*, *P. mirabilis*), Tn5403 (*K. pneumoniae*, *P. mirabilis*), and TnAS3 (*M. morganii*, *P. mirabilis*) insertion elements.

Overall, on the gene level (Figure 2, Figure 3 and Figure 4), *M. morganii, K. pneumoniae*, and *P. mirabilis* include the same region covering four genes: *dsbD*–*trpF*–*ble*–*bla*_NDM-1_. The relative position and orientation of these genes are aligned in the three isolates (same-stranded *trpF*–*ble*–*bla*_NDM-1_, and *dsbD* on the opposite strand). *bla*_NDM-1_ and *ble* are systematically found in adjacent loci and are co-expressed as they share a common promoter upstream: the *bla*_NDM-1_ [6].

The genome similarity of the *bla*_NDM-1_-containing plasmids was further assessed on the genomic level using the average nucleotide identity that was calculated by FastANI 1.3.3. Despite the highly variable lengths, *bla*_NDM-1_-containing plasmids shared common genomic regions of significant identity. *K. pneumoniae* and *P. mirabilis* were less divergent compared with *M. morganii*. The latter was closer to *K. pneumoniae* considering both the overlapping genome length and the identity level. An important confounding factor for all isolates was the incomplete plasmids constructs.

## 3. Discussion

NDM-1 carbapenemase was first reported from Sweden in 2008 from a patient previously hospitalized in New Delhi, India [7]. The encoding gene, however, was already widely disseminated in the Indian subcontinent, and to a lesser extent, in other parts of the world. After its first identification, rapid worldwide spread occurred throughout all continents [8,9] thus placing NDM-1 and its variants among the most successful carbapenemases in terms of epidemiology and dissemination. This is mostly due to the incorporation of *bla*_NDM-1_ in transposable genetic elements and its horizontal transfer by plasmid replicons. Indeed, *bla*_NDM-1_ is commonly detected in conjugative plasmids of various incompatibility groups, through which rapid intra-species and even inter-species spread is possible. In Greece, NDM-1 was first reported in late 2011 [10]. Since then, it has been well-established, together with KPC, VIM, and OXA-48 carbapenemases, as also shown by recent data from our hospital [11]. Unfortunately, during the COVID-19 pandemic there was an increase in carbapenem resistance rates in our institution that was attributed to the increased number of COVID-19 patient admissions, their prolonged time of hospitalization, and the extensive antimicrobial treatment that these patients received. Even worse, the personnel dedicated to infection control were constrained [12].

The bacterial isolates described in this study were recovered by a COVID-19 patient hospitalized during the predominance of the Alpha SARS-CoV-2 variant of concern in our region [13] that caused increased pressure in the national healthcare system. For this reason, the case described here could be characterized as indicative of the outcomes of the concomitant presence of the pandemic with an endemic situation regarding carbapenemases among Gram-negative nosocomial opportunistic pathogens. Before the patient was admitted in the ICU, he was already colonized by a carbapenem-resistant *K. pneumoniae*, whereas, during his ICU stay, he was moreover colonized by a carbapenem-resistant *Acinetobacter baumannii* strain. Among other co-infections for which he received a series of last-line antibiotic treatment, the patient presented blood infection by an NDM-1-producing *K. pneumoniae*. Even though initially discharged from the hospital, he was admitted again after a few months. During this second hospitalization, he presented blood infection with an NDM-1-producing *P. mirabilis* and an NDM-1-producing *M. morganii* resulting in his death by septic shock. Since NDM-1-producing *K. pneumoniae* are common in our hospital but this specific type of carbapenemase is not frequently encountered in *P. mirabilis* and *M. morganii* in our institution, further laboratory investigation was needed to document in vivo transfer.

Plasmids are important “vehicles” for gene transfer among bacteria. Conjugation and mobilization of plasmids may confer clinically relevant traits to host cells, promoting their rapid evolution and adaptation to challenging environments [14]. This is particularly evident for plasmids carrying antimicrobial resistance genes in Enterobacterales like the IncF group (or MOB_F_ according to relaxase typing) [15]. These are the most frequently described conjugative plasmids from human and animal sources and are commonly related to antimicrobial resistance gene transfer and spread [15].

In this study, conjugative plasmids and numerous resistance genes were found in all isolates; interestingly, bioinformatics analyses showed that the *bla*_NDM-1_ and its neighboring genes built a cluster of ordered genes that was present in all three isolates. This cluster was found in IncFIA plasmids of *K. pneumoniae* and *P. mirabilis*, indicating in vivo transfer. The same cluster was found in a plasmid sequence of *M. morganii*, but unfortunately, this specific plasmid could not be characterized regarding its incompatibility group. Thus, a process of conjugation, mobilization of the cluster to another plasmid in *M. morganii*, and then loss of the first plasmid due to fitness costs cannot be excluded. The preservation of the *bla*_NDM-1_-containing cluster was probably favored by the presence of last-line antimicrobials like ceftazidime/avibactam and the versatility of this carbapenemase-encoding gene to incorporate with different types of conjugative plasmid backbones. Indeed, the spread of *bla*_NDM-1_ through IncFIA plasmids is well documented in the literature [16,17,18,19].

A case of in vivo transfer of the *bla*_VIM_ among three different bacterial species in Greece was published in 2012 [20]. To our knowledge, however, this is the first report of three Enterobacterales isolated from the same patient in Greece harboring the *bla*_NDM-1_ with the same surrounding gene cluster located in transferable plasmids. In accordance with our study, recent similar reports from other countries indicate the occurrence of inter-species NDM-1 in vivo transfer. In Portugal, NDM-1-producing *M. morganii* and *P. mirabilis* were isolated from a single patient [21], and the same was reported in Italy for three Enterobacterales: *K. pneumoniae*, *P. mirabilis* and *Enterobacter cloacae*, suggesting a possible transfer of the *bla*_NDM-1_ among clinical species [22]. This in vivo transfer of *bla*_NDM-1_ in hospitalized patients is not limited to species frequently encountered in clinical environments, since it has been reported also among rather less common microorganisms. In the Republic of Korea, this transfer was documented from a *Klebsiella oxytoca* to a *Citrobacter freundii* isolate [23]. In China, an NDM-1-producing *Raoultella ornithinolytica* and an NDM-1-producing *E. cloacae* were isolated from a single patient, indicating in vivo conjugation [24].

This study has a certain limitation. The indications are drawn based on incomplete, non-circular constructs of the plasmids that contain *bla*_NDM-1_. Since the complete reconstruction was not possible, we may have missed even more robust evidence of in vivo transfer or additional information about the structures of the three plasmids.

## 4. Materials and Methods

### 4.1. Hospital Setting and Patient Data

The study was conducted in AHEPA University Hospital, a 700-bed tertiary care hospital in Thessaloniki, Greece that served as one of the reference hospitals for COVID-19 patients in Northern Greece during the pandemic. The isolates described in the present study were collected as part of the standard of care protocol. Patient history data were retrieved by the hospital’s electronic database.

### 4.2. Susceptibility Testing

Bacterial identification and antimicrobial susceptibility testing were performed using the Vitek2 semi-automated system (bioMérieux, Marcy l’Étoile, France). Confirmatory susceptibility testing using the gold standard broth microdilution method was performed where applicable by the MICRONAUT-S MDR MRGN-Screening system (Bruker Daltonics GmbH & Co. KG, Bremen, Germany). The interpretation of susceptibility testing results was done according to the European Committee on Antimicrobial Susceptibility Testing (EUCAST) 2021 clinical breakpoints for bacteria (v 11.0) available at: https://www.eucast.org/ast_of_bacteria/previous_versions_of_documents (accessed on: 30 July 2023).

### 4.3. Whole Genome Sequencing and Genomes Assembly

Libraries were prepared using Ion Torrent technology and Ion Chef workflows (Thermo Scientific, Waltham, MA, USA). Sequencing was performed in the S5XLS system, and analysis of the raw sequencing data was conducted by Ion Torrent Suite v.5.10.0, according to the manufacturer’s instructions. Sequencing of the D1644 isolate performed twice to resolve ambiguities related to the presence of an IncN plasmid type harboring *bla*_NDM-1_. The detection of the plasmid type and downstream analysis for D1644 performed on the merged sequencing products. Raw sequencing data were quality-checked and trimmed to keep only the reads that match the minimum length and quality criteria. Taxonomic assignment was performed as a quality step to filter out samples that may have been contaminated by foreign DNA during sample preparation and to verify the presence of the isolated species. Kraken2 was used for the taxonomy classification built on the standard database that includes NCBI taxonomic information, complete RefSeq microbe genomes, human genomes, and a vector collection [25]. Genomes were de novo assembled by AssemblerSPAdes and SPAdes using the default settings for the k-mers and the Ion Torrent parameter [26].

### 4.4. Functional Annotation of the Assembled Genomes

To identify and annotate genes on the assembled chromosomal and plasmid contigs, we used Prokka v.1.14.6, based on the *Enterococcus* genomic features [27]. Open reading frames (ORFs) were further annotated by manual comparative curation and determination of sequence similarity using the BLASTn searches. The antimicrobial resistant determinants and plasmid types were detected by Staramr version 0.9.1 [28] that integrates diverse molecular profiling tools, including CGE’s Resfinder [29] and PlasmidFinder [30]. To classify contigs either as plasmids or chromosomal, we applied a deep learning methodology called Deeplasmid, that post-assembles plasmids using a combination of assembled sequences and sequence features [31]. In addition, to further verify the synthesis of the plasmid sequences, we used plasmidSPAdes [26] and MOB-Recon [32]. MOB-Recon applies an alternative approach that relies on clustered plasmid reference databases to reconstruct plasmids. MASH indices were calculated to assess the distance of the plasmids against plasmid clusters [33]. ISfinder was used to identify insertion sequence segments in the detected *bla*_NDM-1_-containing plasmids. Nucleotide similarities between genomes were calculated by FastANI [34].

Proksee was used to build circular maps of the *bla*_NDM-1_-containing plasmids [35]. Plasmid sequences were further annotated based on the presence of AMR genes by CARD Resistance Gene Identifier v.5.2.1 [36] and mobile genetic elements by mobileOG-db [37]. *bla*_NDM-1_ genes in the three isolates were examined for the presence of genomic alterations and aligned to assess the evolutionary relationship among isolates. To detect horizontal gene transfer events, we applied an interpolated variable order motifs algorithm [38].

## 5. Conclusions

Based on our molecular microbiology data and the patient’s history, it is probable that in vivo transfer of a *bla*_NDM-1_-containing cluster among three different Enterobacterales may have occurred in our patient in Greece during the COVID-19 pandemic. The treatment options for infections due to carbapenemase-producing microorganisms are limited, and often include formerly abandoned antibiotics or antibiotic combinations. Even these choices, however, depend on the overall patient condition, the antimicrobial susceptibility testing results, and the site of infection. Patients hospitalized for long periods of time can serve as reservoirs of emergence and dissemination of resistance genes. Therefore, this finding highlights the need for close monitoring, patient isolation, implementation of infection control measures, and antimicrobial stewardship, even during difficult times for the national healthcare systems.

## Figures and Tables

**Table 1:** Minimum inhibitory concentrations (MICs) of various antimicrobials for the three study isolates tested with the Vitek2 and the Micronaut-S broth microdilution method (BMD).

MIC (mg/L)/Interpretation												
Antimicrobials	*K. pneumoniae*				*P. mirabilis*				*M. morganii*			
	Vitek2		BMD		Vitek2		BMD		Vitek2		BMD	
Amikacin	_	_	>32	R	-	-	>32	R	8	S	4	S
Amoxicillin/clavulanic acid	≥32	R			≥32	R						
Ampicillin	≥32	R			≥32	R						
Aztreonam	≥64	R			32	R			≤1	S		
Cefepime	≥32	R			16	R			≥64	R		
Cefixime	≥4	R			≥4	R						
Cefotaxime	≥64	R			≥64	R					>2	R
Cefoxitin	≥64	R			≥64	R						
Ceftolozan/tazobactam	≥32	R	>2	R	≥32	R	>2	R			>2	R
Ceftazidime	≥64	R	>128	R	≥64	R	128	R	≥64	R	>128	R
Ceftazidime/avibactam	≥16	R	>4	R	≥16	R	>4	R			>4	R
Ceftriaxone	≥64	R			≥64	R						
Cefuroximeaxetil	≥64	R			≥64	R						
Cefuroxime	≥64	R			≥64	R						
Colistin	≥16	R	>8	R	≥16	R	>8	R	≥16	R	8	R
Ciprofloxacin	≥4	R	>2	R	≥4	R	>2	R	≥4	R	>2	R
Ertapenem	≥8	R			≥8	R						
Fosfomysin	≥256	R	>128	R	≥256	R	>128	R	128	R	128	R
Gentamicin	≥16	R			≥16				2	S		
Chloramphenicol	32	R	>16	R	≥64	R	>16	R			8	S
Piperacillin	_	_	>16	R	_	_	>16	R	≥128	R	>16	R
Piperacillin/tazobactam	≥128	R	>16	R	64	R	>4	I	≥128	R	>16	R
Ticarcillin/clavulanic acid	_	_			_	_			≥128	R		
Ticarcillin	_	_			_	_			≥128	R		
Tobramycin	≥16	R			≥16	R			8	R		
Levofloxacin	≥8	R	>2	R	≥8	R	>2	R			>2	R
Trimeth/sulfamethoxazole	≥320	R	>4/76	R	≥320	R	>4/76	R	≥320	R	>4/76	R
Cefotaxime	≥64	R	>2	R	≥64	R	>2	R				
Tigecycline	_		1	_	_	_	2	_			0.5	
Imipenem	_	_	>8	R	_	_	>8	R	≥16	R	>8	R
Meropenem	_	_	128	R	_	_	32	R	≥16	R	32	R

**Table 2:** Summary statistics of the assembled genomes.

Isolate ID	Species	Genome Length	% Bacteria Abundance	N50	# Contigs
D730	*Klebsiella pneumoniae*	5,772,350	78	169,475	156
D1633	*Proteus mirabilis*	4,256,486	69	174,791	89
D1644	*Morganella morganii*	4,108,218	84	231,582	91

**Table 3:** List of plasmid types per isolate detected by PlasmidFinder. AMR gene: Genes conferring resistance in the corresponding contig; Conf: Confidence level [range: 0–1] of being a true plasmid; Cluster: Closest group of plasmids.

Species	Plasmid Types	Conf/Cluster	AMR Genes
*M. morganii*	lncN	0.555/AA552(I)	-
*P. mirabilis*	Col3 M	0.745/AB434(C)	*qnrD1*
	lncC	1.000/AA860(C)	*-*
	IncFIA(HI1)	1.000/AB187(I)	*bla*_NDM-1_
*K. pneumoniae*	ColRNAI	1.000/AA941(I)	-
	lncC	1.000/AA860(C)	-
	IncFIA(HI1)	0.996/AA964(I)	*bla*_NDM-1_
	IncFIB(K)	0.045/AA275(I)	-

**Table 4:** Genomic features of the *bla*_NDM-1_ plasmids; Conf: Confidence level [range: 0–1] of being a plasmid; Cluster: Closest group of plasmids; OriT: MOB-group to oriT sequences; Type: Plasmid type, Neighbor: Nearest species, MASH dist: Genome-based pairwise distance.

Isolate	Conf	Cluster	OriT	Type	Neighbor	MASH Dist
*K. pneumoniae*	0.996	AA964	MOB_F_	IncFIA	*K. pneumoniae*	0.036
*P. mirabilis*	1.000	AB187	MOB_F_	IncFIA	*K. pneumoniae*	0.016
*M. morganii*	0.304	AA552	-	-	*K. pneumoniae*	0.006

## Data Availability

The data presented in this study are available in the article.
